# Correspondence between alternate measures of maladaptive exercise, and their associations with disordered eating symptomatology

**DOI:** 10.1556/JBA.2.2013.012

**Published:** 2013-08-20

**Authors:** Haidee J. Lease, Malcolm J. Bond

**Affiliations:** ^1^School of Psychology, Flinders University, Adelaide, Australia; ^2^School of Medicine, Flinders University, Adelaide, Australia

**Keywords:** maladaptive exercise, construct validity, disordered eating symptomatology

## Abstract

*Aims*: The study sought to contribute to the measurement of maladaptive exercise by examining the psychometric properties of a variety of instruments and classification algorithms. The primary aim was to identify the items or scales necessary and sufficient to quantify the construct. A secondary aim was to comment on the construct validity of these measures by examining their relationships with disordered eating symptomatology. *Methods*: Questionnaire booklets comprising the Exercise Dependence Scale, the Obligatory Exercise Questionnaire, the Frequency, Intensity, Time Index, and the Eating Attitudes Test were distributed to women attending health and fitness centres. Self-reported age, height, current and desired weight, and length of time as a regular exerciser were also sought. Data were obtained from 302 regular exercisers. *Results*: While there were statistically significant associations among the measures, no two operationalised maladaptive exercise in the same manner. The Frequency-Intensity-Time Index (FIT) was found to be particularly poor. While variation in the size of relationships between maladaptive exercise and disordered eating was noted, all measures were strongly correlated with the exception of FIT, which demonstrated a modest correlation. *Conclusions*: Different conceptualisations of maladaptive exercise have led to alternative operational definitions, resulting in its classification being instrument dependent. Further exploration using samples with differing characteristics (e.g., high/low probability of dependence) may allow more specific recommendations to be made about the optimal measurement of maladaptive exercise. Further, the question of whether maladaptive exercise is more likely a cause or consequence of eating disorders remains.

## Introduction

Participation in physical activity has long been encouraged for its mental and physical benefits (Kirkcaldy & Shephard, 1990; Loumidis & Wells, 1998; Mónok et al., 2012). Due to such benefits, excessive exercise was first thought to be a ‘positive addiction’, with no harmful consequences (Glasser, 1976). However, if taken to extremes, exercise may become physically and psychologically maladaptive (Ackard, Brehm & Steffen, 2002). Morgan (1979) first redefined excessive exercise as a ‘negative addiction’ having observed outcomes such as withdrawal symptoms and depression among runners. Indicators include working out several times every day, or for longer periods than recommended, obsessing over details such as calories expended or heart rate, expressed anger if exercise is interrupted, avoiding social or occupational responsibilities in order to exercise, and centring daily schedules around exercise (Adams & Kirkby, 2002). Terms applied to such behaviour include exercise dependence (Hausenblas & Symons Downs, 2002a), over-exercising (Long, Smith, Midgley & Cassidy, 1993), over-commitment to exercise (Yates, Shisslak, Grago & Allender, 1994), obligatory exercise (Ackard et al., 2002), and exercise addiction (Mathers & Walker, 1999). The term maladaptive exercise is used in the present paper to embrace all of these labels.

The identification of excessive exercisers is often based on characteristics such as time and/or frequency of exercise (Ackard et al., 2002). Alternatively, the meaning ascribed to exercise, or the psychological disposition of the exerciser towards exercise, may differentiate maladaptive exercisers (Davis et al., 1997; Steffen & Brehm, 1999). This may include the motivation driving exercise (Goncalves & Gomes, 2012; Hale, Roth, DeLong & Briggs, 2010; Lejoyeux, Guillot, Chalvin, Petit & Lequen, 2012; Mond & Calogero, 2009; Phelan, Bond, Lang, Jordan & Wing, 2011), or other psychosocial cues such as low self-esteem, high body dissatisfaction, passivity and insecurity, sociocultural pressures, and anxiety (Goncalves & Gomes, 2012; Lejoyeux et al., 2012; McNamara & McCabe, 2012).

Potential physical complications of maladaptive exercise are significant (Cook, Hausenblas & Rossi, 2013), yet injuries are tolerated or denied in order to avoid the discontinuation of exercise. A ‘forced rest’ is met with considerable apprehension (Hausenblas & Symons Downs, 2002b; Mathers & Walker, 1999) and an overwhelming sense of guilt and anxiety over missed exercise sessions (Hubbard, Gray & Parker, 1998). Psychosocial consequences may include impaired relationships, reduced workplace or school performance, and even job loss (Penas-Lledo, VazLeal & Waller, 2002). In short, the promotion of exercise may not necessarily be desirable for individuals for whom exercise has, or may, become an addiction (Allegre, Souville, Therme & Griffiths, 2006; Berczik et al., 2012).

## Measurement of Maladaptive Exercise

Multiple instruments have been developed to evaluate the potential for maladaptive exercise. The Frequency-Intensity-Time Index (FIT; Kasari, 1976) is a quasi-objective measure of physical activity level, and as such is indicative of the purely behavioural assessment of maladaptive exercise alluded to above. As the name suggests the FIT comprises three components: frequency of exercise (i.e., how many times per week), intensity of exercise (i.e., the nature of the activity), and time devoted to exercise (i.e., hours and minutes per day). The product of these components indicates level of physical activity.

The Obligatory Exercise Questionnaire (OEQ; Pasman & Thompson, 1988) measures both quantity of exercise and the psychological meaning of exercise. Results are typically reported in three ways. First, a continuous score may be obtained as a gross indicator of obligation to exercise. Second, respondents can be classified as ‘obligatory exercisers' using a clinical cut-off score (Hubbard et al., 1998; Pasman & Thompson, 1988). Third, subscale scores can be derived on the basis of factor analysis of OEQ items. For example, Ackard et al. (2002) and Steffen and Brehm (1999) report three factors, which differ in item composition. Ackard et al. (2002) labelled their factors exercise fixation, exercise frequency and exercise commitment, while Steffen and Brehm (1999) termed their factors emotional element of exercise, exercise frequency and intensity, and exercise preoccupation. In view of this acknowledged variation, an exploratory factor analysis was first conducted with the current sample, using rigorous rules for the retention of factors, to provide further evidence of the number and nature of constructs inherent in the OEQ.

The Exercise Dependence Scale (EDS; Hausenblas & Fallon, 2002; Hausenblas & Symons Downs, 2002a, 2002b) operationalises exercise dependence according to DSM criteria for substance dependence (American Psychiatric Association, 2000). Seven aspects of dependence are measured: tolerance (the need for increasing amounts of exercise); withdrawal (symptoms such as anxiety are manifest with missed exercise sessions); intention effect (exercise is taken in larger amounts and/or over a longer period than was intended); lack of control (an inability to cut down the amount of exercise); time (frequency/duration of exercise); reductions in, or termination of, other social, occupational or recreational activities because of exercise; and continuance (persistent exercising despite illness or injury that may be exacerbated by the exercise). A total score can be calculated, as can a classification of respondents as exercise dependent, non-dependent but symptomatic, or asymptomatic.

Bamber, Cockerill, Rodgers and Carroll (2003) presented qualitative results suggesting the need for only two diagnostic criteria for exercise dependence. Jointly, impaired functioning (reductions in, or termination of, other activities) in two of psychological, social, occupational, physical, and behavioural domains and withdrawal (either an adverse reaction to exercise interruption or unsuccessful attempts at exercise control) were indicative of exercise dependence. The value of these criteria is the potential for a substantial reduction in the items and subscales required to assess maladaptive exercise without loss of specificity.

Alternate assessments such as those summarised above encompass varying combinations of assessments all deemed relevant to maladaptive exercise. The first aim of the current study was to comment on the efficacy of each by quantifying their level of (dis)agreement. The value of this task is the potential to identify the items or scales necessary and sufficient to quantify the construct of maladaptive exercise.

### Maladaptive exercise behaviour and disordered eating symptomatology

While there is little debate that maladaptive exercise and eating disorders co-exist (Bamber et al., 2003; Costa, Cuzzocrea, Hausenblas, Larcan & Oliva, 2012; Davis et al., 1997; Hubbard et al., 1998), of key interest is whether maladaptive exercise is the primary disorder, or is secondary to eating disorders. If the latter, exercise may be used as a substitute for behaviours such as vomiting or laxative abuse used to expend calories (Bamber et al., 2003). If the former, there is support from studies that note a high level of exercise commonly precedes an eating disorder (Grandi, Clementi, Guidi, Benassi & Tossani, 2011; Penas-Lledo et al., 2002). For example, Davis, Blackmore, Katzman and Fox (2005) noted high levels of exercise in anorexia patients up to one year prior to diagnosis.

Notwithstanding this debate concerning the precise causal link, it is nevertheless clear that the co-existence of the two phenomena allow the use of disordered eating symptomatology in a consideration of the construct validity of the alternate measures of maladaptive exercise included in the current study. It is hypothesised that, regardless of the specific measure or classification, participants with higher levels of maladaptive exercise will also record higher levels of disordered eating symptomatology.

### Summary

The first aim was to contribute to the measurement of maladaptive exercise by exploring the psychometric properties of three instruments. The value lies in the potential to identify both unique and overlapping constructs currently being used to quantify maladaptive exercise. A corollary is an examination of associations between maladaptive exercise and disordered eating symptomatology to allow a commentary on the construct validity of the exercise measures (aim 2). While it was not possible to address causal links between the two, it was possible to establish the joint incidence of the two in a non-clinical sample.

## Methods

### Participants

Eligible participants were women attending one of 10 health and fitness centres located in Adelaide, South Australia, of whom 302 provided a completed questionnaire. Their ages ranged from 18 to 30 years (*M* = 22.3, *SD* = 3.4). On average, participants had been regular exercisers for 7.8 years (*SD* = 5.9 years), with 25% reporting at least 10 years and 10% claiming to have been regular exercisers for at least 15 years. The average BMI of the sample was 23.2 (*SD* = 4.2), with 55 participants (18.3%) classified as underweight (< 20 kg/m^2^) and 72 (24.0%) classified as overweight or obese (>25 kg/m^2^). Participants' desired BMI was significantly lower (*t*(299) = 17.01, *p* < .001; *M* = 20.8, *SD* = 3.1) than their current BMI.

### Procedure

Questionnaires were distributed with permission of the centres' managers. Potential participants were given a verbal briefing of the aims of the study and its requirements. Consent was assumed by the acceptance of a questionnaire. A prepaid envelope was provided for the return of the questionnaire, although sealed collection boxes were also available for participants' convenience. Of all questionnaires distributed, 63% were completed and returned. However, this is likely to be an underestimate due to an indeterminate number of blank questionnaires being inadvertently discarded by centre managers. The study was approved by the Social and Behavioural Research Ethics Committee of Flinders University.

### Questionnaire

Self-reported age, height, current and desired weight, and length of time as a regular exerciser were sought, along with responses to the following scales.

*Obligatory Exercise Questionnaire (OEQ).* The 20-item OEQ (Pasman & Thompson, 1988) was designed to assess attitudes toward exercise routines. Participants respond to each statement as it applies to them using four responses ranging from ‘never true’ to ‘always true’. Items are summed, resulting in a score ranging from 20 to 80, with higher scores representing a stronger perceived obligation to exercise. The total score may also be dichotomised, with participants scoring 50 and above deemed to have a pathological obligation to exercise. Sound reliability and validity of the OEQ have been reported (Coen & Ogles, 1993; Pasman & Thompson, 1988).

*Frequency, Intensity, Time Index (FIT).* A modified version of the FIT (Kasari, 1976) was included as a quasi-objective measure of physical activity level. Each exercise component was assessed using a 1 to 5 scale, with the FIT total being the product of the three component scores (range 1 to 125). Frequency (number of times spent exercising per week) ranged from ‘once a month or less' to 'more than once a day everyday’. Intensity ranged from ‘light aerobic exercise such as normal walking’ to ‘high intensity activities such as running, high impact aerobics and distance cycling’. Time devoted to exercise ranged from ‘up to 30 minutes' to ‘at least 3 hours’. Construct validity information for the FIT has been published (Sharkey, 1997).

*Exercise Dependence Scale (EDS).* The EDS (Hausenblas & Symons Downs, 2002a) measures symptoms of exercise dependence that may lead to social, personal or psychological distress or impairment. Seven aspects of dependence are measured: tolerance, withdrawal, intention effect, lack of control, time, functional impairment, continuance. The 21-item version was used, comprising 3 statements for each of these seven criteria. Participants respond using a 6-point scale (‘never true of me’ to ‘always true of me’), with responses summed to attain individual scale scores ranging from 3 to 18. Respondents can be classified as either exercise dependent (a score of 15 or more on 3 or more subscales), nondependent-symptomatic (a score of 7 to 14 on 3 or more subscales), or asymptomatic (scores of 6 or less on 3 or more subscales). In this form the EDS is highly reliable and valid (Symons Downs, Hausenblas & Nigg, 2004).

*Eating Attitudes Test (EAT-26).* The EAT-26 (Garner & Garfinkel, 1979) is a widely used and highly reliable and valid measure of atypical, disturbed or excessive eating behaviours (Mintz & O’Halloran, 2000). While high scores are not necessarily indicative of a diagnosable disorder, up to 90% accuracy has been noted using the EAT-26 as a screening test for eating disorders in non-clinical samples (Mintz & O’Halloran, 2000). A 6-point response scale is used, with scores being 0 (‘never’, ‘rarely’, 'sometimes’), 1 (‘often’), 2 (‘usually’), or 3 (‘always’). Higher total scores (range 0–78) reflect an increase in disordered eating pathology. A score of 20 or above may also be classified as 'symptomatic’ (Garner & Garfinkel, 1979). Three subscales have been established: dieting (13 items; the likelihood of avoiding fattening or forbidden foods and the preoccupation to be thinner), bulimia and food preoccupation (6 items; fixation with food and likelihood of bulimic episodes), and oral control (7 items; self-control over eating and dietary habits and the perceived pressure to gain weight). All scales provided satisfactory levels of internal consistency, with Cronbach's (1951) a ranging from .68 for bulimia and food preoccupation to .94 for dieting (total score = .93).

## Results

### Obligatory Exercise Questionnaire

Items were first subjected to a principal components analysis. The number of components to retain for varimax rotation was determined using parallel analysis criteria (Lautenschlager, 1989) to take account of both sample size and number of items. An orthogonal rotation was chosen to maximise the variability in derived scales. Two components were supported (48.9% of variance). Within each, items attaining a loading of .50 or greater were considered for scale construction. One component (nine items) was termed ‘Exercise frequency’ (‘I exercise more than three days per week’, ‘I frequently push myself to the limits’). The other (eight items) was termed ‘Exercise fixation’ (‘I have had daydreams about exercising’, ‘When I miss a scheduled exercise session I may feel tense, irritable or depressed’). Descriptive statistics for derived OEQ variables (total score, exercise frequency, exercise fixation) are shown in Table 1. Using the diagnostic cut-off for the OEQ, 108 participants(35.8%) were classified as having a pathological obligation to exercise.

### Frequency, Intensity, Time Index

The FIT provides a single quantification of physical activity that, with the current sample, achieved only a modest level of internal consistency (see Table 1).

**Table 1.** Descriptive data for measures of maladaptive exercise

	Theoretical range	Obtained range	*M*	*SD*	*a*
Obligatory exercise	20–80	22–76	47.1	10.8	.87
Exercise frequency	9–36	10–36	24.8	5.7	.88
Exercise fixation	8–32	8–32	16.3	5.3	.88
FIT index	1–125	1–125	28 0	19.4	.63
Exercise dependence					
Withdrawal	3–18	3–18	8.3	3.5	.89
Continuance	3–18	3–18	7.1	4.0	.93
Tolerance	3–18	3–18	9.7	4.0	.92
Lack of control	3–18	3–18	7.5	4.4	.92
Activity reduction	3–18	3–18	6.0	2.9	.76
Time	3–18	3–18	8.6	4.4	.94
Intention effects	3–18	3–18	7.5	3.2	.93
Withdrawal/activity reduction	6–36	6–36	14.3	5.8	.87

### Exercise Dependence Scale

All EDS subscales offered impressive internal consistency (Table 1). In all cases the mean scores were relatively modest. Using the diagnostic algorithm, only 24 participants(7.9%) were ‘exercise dependent’, with a further 168(55.6%) ‘nondependent-symptomatic’.

### Withdrawal and activity reduction

Following Bamber et al. (2003) a score was computed using the withdrawal and activity reduction (equivalent to functional impairment) EDS subscales (see Table 1). A 'dependent’ classification required a score of at least 15 for both withdrawal and activity reduction, while ‘nondependent-symptomatic’ required scores to be at least 7. This procedure resulted in 2 (0.7%) participants classified as 'dependent’ and 96 (31.8%) ‘nondependent-symptomatic’. In subsequent analyses a single 'symptomatic’ (*n* = 98) classification is used.

### Relationships among alternate classifications of maladaptive exercise

Table 2 presents the relationships among the alternate maladaptive exercise indices introduced above. To allow direct comparison of all indices, both continuous and categorical, all entries are coefficients of strength of association. There were predictably consistent high positive associations among the measures obtained from the OEQ, with a modest level of discrimination between exercise frequency and exercise fixation. The FIT provided the lowest overall levels of association with the other measures. Unsurprisingly, the highest coefficient was with OEQ exercise frequency. Coefficients involving EDS exercise dependence ranged from.45 (FIT) to .74 (OEQ exercise fixation). The latter is notable as these are arguably the most similar constructs measured. The relatively poor association between EDS exercise dependence and OEQ pathological obligation is therefore disappointing. Conversely, withdrawal/activity reduction is strongly associated with OEQ pathological obligation but less related to EDS exercise dependence. To examine whether responses to questions concerning maladaptive exercise could be explained simply in terms of experience with an exercise regimen, the length of time participants had been a regular exerciser was assessed. As shown in Table 2, all coefficients approximated zero.

**Table 2.** Strength of association^†^ among alternate measures of maladaptive exercise

	1	1a	1b	1c	2	3	4
Obligatory exercise	−						
Pathological obligation*	.81^c^	−					
Exercise frequency	.92^c^	.76^c^	−				
Exercise fixation	.91^c^	.74^c^	.70^c^	−			
FIT index	.53^c^	.45^c^	.57^c^	.42^c^	−		
Exercise dependence*	.72^c^	.56^c^	.62^c^	.74^c^	.45^c^	−	
Withdrawal/activity reduction*	.62^c^	.78^c^	.53^c^	.63^c^	.33^c^	.60^c^	
Years as a regular exerciser	.06	.01	.06	.01	−.01	.03	−.01

*Notes: N* = 302 for all analyses; * categorical classifications; ^c^ *p* < .001; ^†^ Pearson's *r* between continuous measures, ϕ (phi) between categorical measures, η (eta) between a continuous and a categorical measure.

Relationships among the three categorical classifications of maladaptive exercise were considered in greater detail by examining pairwise agreement between them. All participants classified ‘at risk’ by the EDS were also considered to have a pathological obligation to exercise using the OEQ. However, when the EDS ‘at risk’ and 'symptomatic’ participants were combined, there was only 68.2% overall agreement between the measures. The mismatch was predominantly due to participants classified 'symptomatic’ (EDS) who recorded no pathological obligation to exercise using the OEQ. The pairing of EDS and withdrawal/activity reduction again produced only modest agreement (68.9%) when EDS ‘at risk’ and 'symptomatic’ participants were combined. While no ‘asymptomatic’ participant was diagnosed as exercise dependent, only 51.0% of those classified ‘at risk’ or 'symptomatic’ received a diagnosis of exercise dependence using the withdrawal/activity reduction measure. Comparing the OEQ classification with with-drawal/activity reduction produced a better level of agreement (82.1%). The disagreement in classification between these two measures was in both directions, with 10.6% of participants with an obligation to exercise (OEQ) considered ‘normal’ (withdrawal/activity reduction) and 7.3% with no obligation to exercise diagnosed as exercise dependent.

**Table 3.** Strength of association^†^ between measures of maladaptive exercise and disordered eating symptomatology

	EAT-26 total score	EAT-26 symptomatic^*^	EAT-26 dieting	EAT-26 bulimia	EAT-26 oral control	Desired BMI
Obligatory exercise	.67^c^	.59^c^	.67^c^	.48^c^	.48^c^	−.32^c^
Pathological obligation^*^	.60^c^	.55^c^	.59^c^	.42^c^	.43^c^	−.26^c^
Exercise frequency	.55^c^	.50^c^	.55^c^	.34^c^	.45^c^	−.18^b^
Exercise fixation	.74^c^	.63^c^	.74^c^	.59^c^	.46^c^	−.43^c^
FIT index	.34^c^	.26^c^	.34^c^	.32^c^	.16^b^	−.14^a^
Exercise dependence^*^	.60^c^	.46^c^	.59^c^	.46^c^	.40^c^	−.32^c^
Withdrawal/activity reduction^*^	.58^c^	.49^c^	.58^c^	.45^c^	.38^c^	−.36^c^
Years as a regular exerciser	−.10	.06	−.11^a^	−.05	−.09	.16^b^

*Notes: N* = 302 for all analyses except Desired BMI (N = 300); ^*^ categorical classifications; ^a^ *p* < .05; ^b^ *p* < .01; ^c^ *p* < .001; ^†^ Pearson's *r* between continuous measures, ϕ (phi) between categorical measures, η (eta) between a continuous and a categorical measure.

### Relationships between maladaptive exercise and disordered eating symptomatology

Finally, measures of maladaptive exercise were examined for their associations with measures of disordered eating symptomatology (Table 3). Included are the total score from the EAT-26, the three EAT-26 factor scores, and the EAT-26 symptomatic classification. There were 75 (24.8%) symptomatic participants. Desired BMI relative to current BMI is also included. As in Table 2 entries are coefficients of strength of association. While most coefficients are indicative of very significant statistical associations, it is the size of the relationships that is equally if not more informative in this situation. The FIT index can be highlighted for its particularly modest associations with all indices of disordered eating symptomatology. However, there appears little to distinguish between the other measures. Of some note are the (slightly) stronger associations when continuous rather than categorical variables are employed, in accord with statistical theory (Cohen, 1983). Of additional note are the consistently low associations between desired BMI and maladaptive exercise, while years as a regular exerciser was similarly not predictive of disordered eating.

## Discussion

The primary aim was to address the acknowledged lack of consistency in maladaptive exercise measurement (Davis et al., 1997; Hausenblas & Symons Downs, 2002b; Mónok et al., 2012). The FIT Index was an early example of such a measure, while the Exercise Dependence Scale (EDS) and the Obligatory Exercise Questionnaire (OEQ) were also used. An algorithm based on DSM criteria, suggesting that Withdrawal and Impaired Functioning (Activity Reduction), if jointly indicated, determine maladaptive exercise, was also tested. In total this provided seven measures (four continuous and three categorical) for which both intra-associations and inter-associations with disordered eating symptomatology were examined. Based on these analyses it is difficult to definitively conclude which measure or measures provides the most reliable, valid and parsimonious commentary on maladaptive exercise. Nevertheless, the following observations arise from the data presented.

The first contribution of the current study is a more definitive statement regarding the number of factors inherent in the OEQ. Previously, three factors have been suggested, represented by differing items and names. In the current study the application of more cautious rules for the retention of factors resulted in only two scales, with the unequivocal labels of exercise frequency and exercise fixation. These scales clearly distinguish between the purely behavioural act of exercising and the more affective elements associated with exercising. Exercise fixation as described in the current study subsumes ‘exercise fixation’ and ‘exercise commitment’ (Ackard et al., 2002) and also 'the emotional element of exercise’ and ‘exercise preoccupation’ (Mónok et al., 2012; Steffen & Brehm, 1999).

The routine assumption that factors with an eigenvalue of at least one are non-random and therefore meaningful is a common error that often results in factors that are different in nuance only. While retaining more factors does increase the variance accounted for by a factor model, the risk is the promulgation of constructs that are not genuinely unique and may not generalise to other samples (cf. Ackard et al., 2002; Steffen & Brehm, 1999). In this spirit it remains important that the two proposed OEQ factors are replicated in other research settings to determine whether they generalise beyond the current sample.

The poorest measure in the current study was the FIT index. This is in accord with its purely behavioural assessment of activity. This metric has not previously been used often and the recommendation on the basis of the current data is that it offers little that cannot be established using the more common indices included here. Given its focus on frequency, intensity and duration it is perhaps not surprising that the FIT index was most closely associated with OEQ exercise frequency. Yet even then only a modest association was noted.

Greater attention may need to be given to the algorithm applied to the EDS to determine 'dependent’ and ‘non-dependent-symptomatic’ individuals. Notwithstanding the fact that the constructs evaluated by the EDS are in accord with DSM criteria for dependence (Costa et al., 2012), given that high scores need to be recorded for only three of the seven subscales, it is possible for these classifications to be made on the basis of entirely differing, or at best overlapping, clusters of symptoms. Hypothetically, one person may score above 15 for tolerance, withdrawal and functional impairment, while another may score above 15 for intention effect, lack of control and time. That is, different profiles of dependence are possible, with both termed 'dependent’. Yet the context of maladaptive exercise for these two individuals (e.g., antecedents, consequences) may be quite dissimilar. Severity, which may also dictate the treatment that may be most beneficial, could also depend on the profile of high scores. This is not currently acknowledged in the use of the EDS. Future research may benefit from analysing differences between exercise dependent individuals who have scored highly on different EDS subscales in order to determine the importance of such variation.

A corollary to this argument is that the diagnosis based on withdrawal/activity reduction is also a subset of the possible combinations available from the EDS, although using two rather than three subscales. Perhaps, as Bamber et al. (2003) have argued, a specified minimal subset of information is all that is required to establish maladaptive exercise. It is true that among the alternative measures reviewed, and in relation to the measures of disordered eating, the with-drawal/activity reduction performed on a par with longer, more detailed assessments. Of note is the high level of agreement discussed above between withdrawal/activity reduction and OEQ pathological obligation to exercise (82.1%).

Of course, withdrawal/activity reduction as used in the current study is not a direct application of the guidelines offered by Bamber et al. (2003). Their determination was based on qualitative interviews, while its operationalisation in the current study was based on available quantitative data. To allow this, activity reduction was substituted for impaired functioning. While it appears that these terms are essentially equivalent, they may not be synonymous symptoms. It may be prudent in future studies to consider a more literal quantitative measurement of withdrawal and functional impairment in this context.

Ultimately, scale choice may be guided by whether a diagnostic or screening test is desired. Without a ‘gold standard’ for either the diagnosis of, or a screening assessment of, maladaptive exercise it is difficult to evaluate the degree to which the reviewed measures fulfil these roles. However, given the different goals of each class of test (minimisation of false negatives for a screening test, minimisation of false positives for a diagnostic test), it is appropriate to consider the nature of at least the categorical variables reviewed. For example, it may be postulated that OEQ pathological obligation to exercise provides only a screening assessment due to its relatively low cut-off score. Conversely, EDS exercise dependence offers provision for both a diagnostic cut-off (dependent) and a screening cut-off (non-dependent symptomatic). Finally, from the description given by Bamber et al. (2003), the withdrawal/activity reduction classification is also diagnostic as it is based on DSM criteria.

The above discussion does not entirely explain the modest level of agreement between the categorical variables considered in the current study. Even as screening instruments, a reasonable overlap in classification would be expected. Perhaps the nature of the current sample militated against the performance of some of the scales. For example, relatively few participants were diagnosed as exercise dependent (*n* = 2 for withdrawal/activity reduction). The application of these instruments in populations with a higher proportion of dependent individuals may result in a higher level of agreement.

The current study provided support for the noted association between eating disordered symptomatology and maladaptive exercise (Bamber et al., 2003; Costa et al., 2012; Davis et al., 1997; Hubbard et al., 1998). Reasonably strong associations were found for all maladaptive exercise measures, with the exception of the FIT index which, nevertheless, was moderately associated. Conversely, a low association was found between years as a regular exerciser and disordered eating, providing further evidence that maladaptive exercise is more than the absolute amount of exercise undertaken (Ackard et al., 2002). Also, a consistently low association emerged between desired BMI and maladaptive exercise. It is possible that the desire to change weight may not be important to all maladaptive exercisers. However, indifference about weight may not equate to body satisfaction, as body shape can be equally important (Hale et al., 2010). Future research may wish to explore this relationship further. Moreover, as weight change does not appear to be the motive for exercise among participants, it is possible that maladaptive exercise is exhibited as a primary disorder, leading to disordered eating (Grandi et al., 2011). The adjunct measurement of exercise motives in future research would usefully address this possibility.

Maladaptive exercise is a phenomenon currently conceived and measured differently by different researchers. Such alternatives result in a variety of interpretations of dependence. Unified measurement would no doubt serve to progress the understanding of this condition. Before definitive comments about measurement can be made, however, it is recommended that comparisons such as those reported are made using samples of varying composition. Indeed, the current data were obtained only from an opportunistic community sample. Further, research would benefit from maladaptive exercise definitions being compared to overt exercise levels. The lack of a formal measure of exercise behaviour in the current study was a significant limitation. Nevertheless, the data presented has provided a useful contribution to the debate concerning the measurement of maladaptive exercise.
